# Role of *parC* Mutations at Position 84 on High-Level Delafloxacin Resistance in Methicillin-Resistant *Staphylococcus aureus*

**DOI:** 10.3390/antibiotics13070641

**Published:** 2024-07-11

**Authors:** Silvia Bolaños, Cesar Acebes, Óscar Martínez-Expósito, José Antonio Boga, Javier Fernández, Carlos Rodríguez-Lucas

**Affiliations:** 1 Servicio de Microbiología, Hospital Universitario Central de Asturias (HUCA), Avenida de Roma s/n, 33011 Oviedo, Spain; silviamaria.bolanos@sespa.es (S.B.); cesar.acebes@sespa.es (C.A.); oscar.martinez@sespa.es (Ó.M.-E.); joseantonio.boga@sespa.es (J.A.B.); fernandezdjavier@uniovi.es (J.F.); 2 Grupo de Microbiología y Patología Infecciosa, Instituto de Investigación Sanitaria del Principado de Asturias (ISPA), 33011 Oviedo, Spain; 3 Research & Innovation, Artificial Intelligence and Statistical Department, Pragmatech, 33001 Oviedo, Spain; 4 Departamento de Biología Funcional, Universidad de Oviedo, 33006 Oviedo, Spain

**Keywords:** delafloxacin, fluoroquinolone, *Staphylococcus aureus*, MRSA, *parC*, QRDR

## Abstract

High-level delafloxacin-resistant (H-L DLX-R) *Staphylococcus aureus* isolates (minimum inhibitory concentration ≥1 mg/L) associated with mutations affecting position 84 of ParC have emerged. We aimed to elucidate the role of these mutations as a mechanism of H-L DLX resistance in methicillin-resistant *S. aureus* (MRSA) isolates recovered from blood cultures. Susceptibility to DLX was determined in 75 MRSA isolates by E-test, and an rt-PCR was developed to detect mutations affecting position 84 of ParC to screen a further 185 MRSA isolates. The genomes of 48 isolates, including all DLX-R isolates or with alterations at position 84, and also a subset of DLX-susceptible isolates were analyzed. Among the 75 isolates studied, 77.34% were DLX-susceptible and only 4 H-L DLX-R isolates were found. Seven (3.8%) isolates with alterations at position 84 of ParC were detected by rt-PCR. Genomic analysis showed that 89.9% (8/9) of isolates with the substitution E84K/G in ParC, together with other mutations in *gyrA* and *parC*, were H-L DLX-R. However, the E84K substitution in ParC alone or with other alterations was found in two isolates without H-L DLX-R. Alterations at position 84 of ParC are rare but play a key role in H-L DLX resistance in MRSA but only when other alterations in GyrA are present.

## 1. Introduction

Fluoroquinolones are a family of broad-spectrum antimicrobials frequently used in human medicine to treat a variety of infections caused by a wide range of Gram-positive and Gram-negative microorganisms worldwide. However, their continuous use since the late 1980s has resulted in increased resistance to these drugs [1]. Resistance to fluoroquinolones is particularly relevant in the treatment of outpatients or bone and joint infections, where their high bioavailability makes them a good alternative when other options cannot be used. Recently, delafloxacin (DLX), a novel anionic fluoroquinolone, has been approved by the Food and Drug Administration (FDA) for the treatment of acute bacterial skin infections and skin structure infections (ABSSSIs) and community-acquired pneumonia (CAP) [2]. DLX exhibits a broad spectrum of activity against Gram-negative and Gram-positive bacteria, including methicillin-resistant *Staphylococcus aureus* (MRSA). Most fluoroquinolones have a binding affinity for DNA gyrase in Gram-negative microorganisms or for topoisomerase IV in Gram-positive microorganisms, whereas DLX has an equal affinity to bind both enzymes [2]. This dual targeting ability has been associated with an increased activity against Gram-positive bacteria, including ciprofloxacin- and levofloxacin-resistant isolates, and may contribute to a low frequency of mutant selection. DLX has demonstrated potent in vitro antimicrobial activity against MRSA isolates, showing at least 8-fold greater activity than levofloxacin or moxifloxacin [3,4,5,6,7,8,9]. Since most MRSA isolates are resistant to fluoroquinolones, the recovered activity of DLX opens a new alternative for the treatment of infections caused by this pathogen [2]. Nevertheless, some recent studies have described the emergence of DLX resistance among MRSA strains, including isolates with high levels of resistance [minimum inhibitory concentration (MIC) ≥1 mg/L] to this drug in a small number of them [6,7,8,9,10]. Resistance to DLX in *S. aureus* is mainly mediated by the acquisition of multiple and simultaneous mutations in quinolone resistance-determining regions (QRDRs) in both targets, namely DNA gyrase and topoisomerase IV. Of particular interest are mutations affecting position 84 of the ParC protein (E84K/G/V), which have been suggested to play a key role in high-level DLX resistance in *S. aureus* [2,3,4]. Although high-level DLX resistance in *S. aureus* is rare, the few isolates with this level of resistance that have been reported to date have shown mutations affecting position 84 of ParC, while isolates with lower MICs to DLX have not shown mutations affecting this position [2]. 

In this work, we aimed to elucidate the role of mutations affecting position 84 of the ParC protein as a mechanism of high-level resistance to DLX in MRSA isolates. We performed a DLX susceptibility study against a collection of MRSA isolates and developed a real-time PCR (rt-PCR) to detect mutations affecting position 84 of ParC. Subsequently, we utilized this assay to analyze a separate collection of MRSA isolates. To determine the role of mutations affecting position 84 of ParC, the genomes of isolates with and without mutations were sequenced and analyzed.

## 2. Results

### 2.1. Delafloxacin Susceptibility Results and Genomic Traits of Isolates 

Among the 75 MRSA isolates included in the DLX susceptibility study, 72 were resistant (MICs > 1 mg/L) and 3 of them were susceptible with increased exposure (MICs > 0.001 mg/L–≤1 mg/L) to ciprofloxacin and levofloxacin based on the European Committee of Antimicrobial Susceptibility Testing (EUCAST) breakpoints [11]. The MIC_50_ and MIC_90_ for DLX of the ciprofloxacin- and levofloxacin-resistant MRSA isolates were 0.25 mg/L and 0.5 mg/L, respectively, with a MIC range between 0.125 and 3 mg/L. The three isolates categorized as susceptible with increased exposure to ciprofloxacin and levofloxacin had MICs to DLX of 0.003, 0.006, and 0.008 mg/L. Fifty-eight of the total isolates (77.34%) were categorized as susceptible to DLX according to the EUCAST breakpoint for skin and skin structure infections (S ≤ 0.25 mg/L) [11]. Thus, 17 of the total isolates (22.67%) were categorized as resistant to DLX, 4 of which showed high levels of resistance (MIC > 1 mg/L). On the other hand, the only three (4.0%) isolates susceptible with increased exposure to ciprofloxacin and levofloxacin were categorized as susceptible to DLX according to the EUCAST breakpoint for CAP (S ≤ 0.016 mg/L). Genomic analysis of the 17 DLX-resistant and 24 DLX-susceptible MRSA isolates selected revealed multiple mutations in the QRDRs affecting the GyrA, ParC, and/or ParE proteins (Table 1). 

All ciprofloxacin- and levofloxacin-resistant isolates sequenced (39) had the substitution S84L in GyrA and the substitution S80F/Y in ParC, regardless of their MICs to DLX. However, all the isolates with high-level resistance to DLX had the above substitutions plus mutations affecting position 84 of ParC (substitutions E84K/G), substitutions not found in the isolates with MICs to DLX < 1 mg/L. Finally, isolates with substitutions in ParE (D432N and P585S) showed MICs to DLX similar to isolates without these alterations. 

### 2.2. Sensitivity and Specificity of the Developed Real-Time PCR

The designed rt-PCR was able to discriminate correctly between wild-type (WT) and mutant alleles in all isolates analyzed (sensitivity and specificity of 100%) in their validation performed with 37 WT isolates, 3 isolates with the E84K substitution, and 1 with the E84G substitution in ParC. Isolates with a WT phenotype or the E84K substitution were directly detected by FAM (6-carboxyfluorescein fluorescent dye) and VIC (2′-chloro-7′phenyl-1,4-dichloro-6-carboxy-fluorescein fluorescent dye) probe signals, respectively, while the only isolate with the E84G substitution did not show any signal with either the FAM or VIC probes, and further molecular studies were needed to characterize it.

### 2.3. Prevalence and Genomic Traits of Isolates with Mutations Affecting Position 84 of ParC

Screening with the designed rt-PCR produced results that were compatible with mutations affecting position 84 of ParC in 7 of the 185 (3.8%) isolates analyzed. Genomic analysis of these isolates confirmed the presence of mutations affecting position 84 of ParC as follows: five isolates with the E84K substitution, one with the E84G substitution, and one with the E84Q substitution (Table 2). MICs to ciprofloxacin, levofloxacin, and DLX of the seven isolates and their mutations in the QRDRs detected by genomic analysis are shown in Table 2. 

Four of the seven isolates exhibited high-level DLX resistance with a different QRDR mutation profile: three of them had the S84L substitution in GyrA, E84K and S80F in ParC, and were WT (n = 1) or had the P456S substitution in ParE (n = 2); and a single isolate had the S84L and S85P substitutions in GyrA plus E84G and S80Y in ParC. In contrast, one isolate with the same QRDR mutation profile as an isolate with high-level DLX resistance (S84L substitution in GyrA, and E84K and S80F in ParC) had a MIC of 0.5 mg/L to DLX. Another isolate with a MIC of 0.38 mg/L to DLX had the E84Q substitution, which has not previously been associated with quinolone resistance. Finally, an isolate with only the E84K substitution in ParC and no other mutations in QRDRs was susceptible to DLX (MIC 0.006 mg/L) and levofloxacin (increased exposure).

## 3. Discussion

In the first step of this work, we evaluated the activity of DLX against a collection of 75 MRSA isolates. Despite the fact that most of the isolates studied were resistant to ciprofloxacin and levofloxacin, DLX showed great activity, with 77.34% of the isolates classified as susceptible to this drug according to the EUCAST breakpoint for skin and skin structure infections. In a previous study, Iregui et al. found a similar rate of susceptibility to DLX (78%) in a collection of 279 MRSA isolates [10]. Similarly, the MIC_50_ and MIC_90_ for DLX of the ciprofloxacin- and levofloxacin-resistant MRSA isolates included in our work (0.25 and 0.5 mg/L, respectively) are also consistent with previous studies that have reported a range of 0.06 to 0.25 mg/L and 0.25 to 1 mg/L for MIC_50_ and MIC_90_ in MRSA isolates, respectively [2]. This high activity of DLX in fluoroquinolone-resistant isolates has previously been associated with the low impact of the most commonly observed mutations in these isolates, conducing to the S84L substitution in GyrA and the S80F/Y substitution in ParC. In this regard, we found 22 isolates with the aforementioned double alterations in GyrA and ParC, with or without alterations in ParE (D432N and P585S), categorized as susceptible to DLX, and 13 isolates with the same mutations and MICs to DLX in the range of 0.25 to 0.5 mg/L categorized as DLX-resistant. However, in a previous ABSSSI Phase III clinical trial, a microbiological response rate of 98.6% was observed in patients with infections due to *S. aureus* isolates with these double mutations and MICs to DLX below to 0.5 mg/L [6]. Therefore, these mutations do not appear to be associated with clinical failure in ABSSSIs treated with DLX. On the other hand, a recent study has observed that mutations affecting position 84 of ParC may be associated with high levels of DLX resistance (MICs ≥ 1 mg/L) [4]. The authors found E84K/V substitutions in ParC exclusively in four isolates with high levels of DLX resistance but not in other isolates with lower MICs to DLX [4]. However, only a few isolates with these MICs have been previously reported and sequenced. Thus, previous studies reporting isolates with mutations affecting position 84 of ParC included a single isolate with a MIC to DLX of 4 mg/L that had the S84L and E88K substitutions in GyrA plus the S80Y and E84G substitutions in ParC [6]; four isolates with MICs to DLX > 2 mg/L that had the S84L substitution and E88K or S85P in GyrA plus the S80F/Y and E84G substitutions in ParC [10]; and finally, one isolate with a MIC of 1 mg/L to DLX that had the S84L substitution in GyrA plus the S80Y and E84G substitutions in ParC [5]. To the best of our knowledge, the present study has analyzed the largest collection (n = 11) of isolates with mutations affecting position 84 of ParC. This could be attributed to screening performed with rt-PCR, which allows rapid and reliable detection of these mutations in large collections of isolates. Our results support the hypothesis that mutations affecting position 84 of ParC are key to the high levels of resistance to DLX in *S. aureus*. In fact, eight of the nine (88.89%) isolates that had the substitution E84K/G in ParC along with mutations in *gyrA* showed high levels of resistance to DLX. In contrast, a single isolate, despite having the same QRDR mutations profile, showed a MIC of 0.5 mg/L for DLX. This finding was only reported by Nilius et al., who found two *S. aureus* isolates with MICs to DLX below 1 mg/L that had changes in Ser84 of ParC. However, the authors did not specify the specific amino acid changes present [3]. Therefore, their results can be explained by an amino acid change that does not affect fluoroquinolone activity. In this regard, we found an isolate with E84Q and S80F substitutions in ParC plus the S84L substitution in GyrA that had a MIC to DLX of 0.38 mg/L. The E84Q substitution has not been previously associated with fluoroquinolone resistance, so the ciprofloxacin, levofloxacin, and DLX resistance of the isolate with this substitution could by mediated by the other two substitutions described above in the GyrA and ParC proteins. Lastly, we found an isolate with a MIC against DLX of 0.006 mg/L that only had the E84K substitution in ParC but did not have mutations associated with fluroquinolone resistance in the *gyrA*, *gyrB*, or *parE* genes. This finding, consistent with the dual targeting ability of DLX, highlights that although mutations affecting position 84 of ParC are associated with high MICs to DLX, this could only occur if the isolates also had mutations in the *gyrA* gene. The main limitations of our study were the small sample size and the lack of quality control strains in the DLX susceptibility study. Moreover, we did not evaluate the recently described contribution of efflux pumps [4] to DLX resistance in our isolates.

## 4. Material and Methods

### 4.1. Bacterial Strains

Two frozen isolate collections of seventy-five isolates and one hundred and eighty-five MRSA clinical isolates were included in the study. All of them had been previously recovered from blood culture samples at two Microbiology Services of the Hospital Universitario de Cabueñes (Gijón, Spain) and Hospital Universitario Central de Asturias (Oviedo, Spain). Identification and antimicrobial susceptibility testing of isolates were previously performed as part of the routines of the two Microbiology Services, using MALDI-TOF MS (Bruker Daltonik GmbH, Bremen, Germany) and the MicroScan system (Beckman Coulter, Brea, CA, USA), respectively. MIC results were interpreted according to the EUCAST breakpoints [11].

### 4.2. Delafloxacin Susceptibility Study

MICs for DLX were determined in 75 MRSA isolates from the first collection using gradient strips (Liofilchem, Roseto degli Abruzzi, Italy) following the manufacturer’s instructions. MIC results were interpreted according to the EUCAST breakpoint for skin and skin structure infections for DLX (S ≤ 0.25 mg/L; R > 0.25 mg/L) [11]. Isolates with MICs ≥ 1 mg/L for DLX were categorized as high-level resistant and were analyzed in depth. The genomes of 41 isolates were sequenced, including all the DLX-resistant isolates (17), and also 24 DLX-susceptible isolates, which were later used as controls for evaluation and screening with the rt-PCR developed in this study (see below).

### 4.3. Development and Validation of a Real-Time PCR Allelic Discrimination Assay for the Detection of Mutations Affecting Position 84 of ParC

An rt-PCR assay was developed to discriminate between the WT and E84K mutant alleles of *parC*. For this purpose, oligonucleotides specific for amplification of a fragment of the *parC* gene (PARC-F: GGTCAATATCATCCACATGGAGACT; and PARC-R: GTCAAGACTGGAAGTTACGACATGTC) and two TaqMan minor groove binder (MGB) probes (ThermoFisher, Waltham, MA, USA) labeled with FAM (PARC-E84-FAM: TGTACGAAGCAATGGTC; for WT allele detection, recognizing GAA) and VIC (PARC-E84K-VIC: GTGTACAAAGCAATGGTC; for detection of the allele with the E84K mutation, recognizing AAA) were designed using Primer Express^TM^ v3.0.1 software (Applied Biosystems, Waltham, MA, USA). The rt-PCR mixture was prepared as follows: 5 µL of Brilliant III Ultra-Fast QPCR Master Mix (Agilent Technologies, Santa Clara, CA, USA), 1 µL of primers and probes mix (with each primer at 4 µM and each probe at 1 µM), and 4 µL of boil-extracted DNA to a final volume of 10 µL. The cycling conditions were as follows: 95 °C for 10 min; and 40 cycles of 95 °C for 5 s and 70 °C for 30 s. The sensitivity and specificity of the rt-PCR were assessed using the 41 isolates previously characterized (see Section 4.2) at the molecular level (37 WT, 3 with the E84K substitution, and 1 with the E84G substitution in ParC). 

### 4.4. Screening of MRSA Isolates with Mutations Affecting Position 84 of parC with the rt-PCR Developed

Isolates belonging to the second collection of MRSA isolates (n = 185) were first analyzed by the rt-PCR designed in this study to detect isolates with mutations affecting position 84 of ParC, and only isolates with mutations detected by this assay were subjected to DLX susceptibility testing and genome sequencing, as previously described. Isolates with known substitutions at position 84 of ParC (E84K and E84G) and WT isolates were used as positive and negative controls for rt-PCR.

### 4.5. Whole Genome Sequencing (WGS) and Bioinformatic Analysis

The genomes of 17 DLX-resistant and 24 DLX-susceptible MRSA isolates detected in the delafloxacin susceptibility study, and 7 MRSA isolates with mutations affecting position 84 of ParC detected by rt-PCR were sequenced using the Illumina platform at Eurofins Genomics (Ebersberg, Germany), and a bioinformatic analysis was performed. Genomic DNA was extracted with the GenElute Bacterial Genomic DNA Kit (Sigma-Aldrich; Merck Life Science, Madrid, Spain) following the manufacturer’s instructions. Paired-end reads of 150 bp were sequenced on a NovaSeq 6000. The obtained raw reads were assembled using SPAdes or the VelvetOptimiser.pl script implemented in the “on line” version of PLACNETw (https://openebench.bsc.es/tool/placnetw, accessed on 23 June 2023) [12]. The identification of resistance determinants was performed in silico using the online tool ResFinder 4.1 from the Center for Genomic Epidemiology of the Technical University of Denmark [13,14].

## 5. Conclusions

A high proportion (77.34%) of fluoroquinolone-resistant MRSA isolates with mutations in the *gyrA* and *parC* genes were found to be susceptible to DLX. Therefore, it may provide a new option for the treatment of infections caused by these multidrug-resistant microorganisms. In particular, DLX may be an excellent sequential therapy for bone and joint infections caused by microorganisms resistant to other fluoroquinolones, as is often the case with MRSA isolates. Mutations affecting position 84 of the ParC protein are rare (3.8%) in *S. aureus* but play a key role in high levels of resistance to DLX but only when they are associated with other mutations in the *gyrA* gene. The high MICs to DLX conferred by this QRDR mutation profile may limit the use of this drug in clinical practice. Surveillance and genomic analysis of isolates with high MICs to DLX should be performed to characterize their mechanism of resistance and evaluate their impact on the efficacy of this new antimicrobial. In this regard, the rt-PCR developed here may provide a rapid and reliable assay to detect mutations affecting position 84 of the ParC protein.

## Figures and Tables

**Table 1:** Fluoroquinolone susceptibility profile and QRDR analysis for the 41 sequenced methicillin-resistant *Staphylococcus aureus* isolates obtained from the delafloxacin susceptibility study.

No. of Isolates	MIC (mg/L)			QRDR Mutation Profile			
	DLX	CIP	LVX	GyrA	GyrB	ParC	ParE
1	0.003	≤1	≤1	WT	WT	WT	WT
1	0.006	≤0.5	≤1	WT	WT	WT	WT
1	0.125	>2	4	S84L	WT	S80F	WT
5	0.19	>2	>4	S84L	WT	S80F	WT
13	0.25	>2	>4	S84L	WT	S80F	WT
2	0.25	>2	>4	S84L	WT	S80F	D432N
1	0.25	>2	>4	S84L	WT	S80Y	P585S
4	0.38	>2	>4	S84L	WT	S80F	WT
2	0.38	>2	>4	S84L	WT	S80F	P585S
1	0.38	>2	>4	S84L	WT	S80F	D432N
4	0.5	>2	>4	S84L	WT	S80F	WT
1	0.5	>2	>4	S84L	WT	S80F	P585S
1	0.5	>2	>4	S84L	WT	S80F	D432N
3	1.5	>2	>4	S84L	WT	**E84K**; S80F	WT
1	3	>2	>4	S84L	WT	**E84G**; S80F	WT

MIC: minimum inhibitory concentration; QRDR: quinolone resistance-determining region; DLX: delafloxacin; CIP: ciprofloxacin; LVX: levofloxacin; WT: wild type. Mutations affecting position 84 of the ParC protein are shown in bold.

**Table 2:** Fluoroquinolone susceptibility profile and QRDR analysis for the seven methicillin-resistant *Staphylococcus aureus* isolates detected by real-time PCR screening.

No. of Isolates	MIC (mg/L)			QRDR Mutation Profile			
	DLX	CIP	LVX	GyrA	GyrB	ParC	ParE
1	0.006	2	≤1	WT	WT	**E84K**	WT
1	0.38	>2	>4	S84L	WT	**E84Q**; S80F	WT
1	0.5	>2	>4	S84L	WT	**E84K**; S80F	WT
1	1.5	>2	>4	S84L	WT	**E84K**; S80F	WT
1	1.5	>2	>4	S84L	WT	**E84K**; S80F	P456S
1	2	>2	>4	S84L	WT	**E84K**; S80F	P456S
1	6	>2	>4	S84L; S85P	WT	**E84G**; S80Y	WT

MIC: minimum inhibitory concentration; QRDR: quinolone resistance-determining region; DLX: delafloxacin; CIP: ciprofloxacin; LVX: levofloxacin; WT: wild type. Mutations affecting position 84 of the ParC protein are shown in bold.

## Data Availability

The original contributions presented in the study are included in the article. Further inquiries can be directed to the corresponding author.
